# Schmerzbedingte Stigmatisierung bei Patienten mit Brust‑, Darm‑, Prostata- oder Lungenkrebs: Ergebnisse einer bizentrischen registerbasierten Querschnittstudie

**DOI:** 10.1007/s00482-023-00752-3

**Published:** 2023-09-14

**Authors:** A. Roicke, P. Esser, B. Hornemann, J. Ernst

**Affiliations:** 1 Abteilung Medizinische Psychologie und Medizinische Soziologie, Universitätsklinikum Leipzig AöR, Philipp-Rosenthal-Str. 55, Haus W, 04103 Leipzig, Deutschland; 2 Universitäts KrebsCentrum (UCC), Psychoonkologie, Universitätsklinikum Carl Gustav Carus, Dresden, Deutschland

**Keywords:** Depression, Angst, Lebensqualität, Psychoonkologie, Neoplasie, Depression, Anxiety, Quality of life, Psycho-oncology, Neoplasm

## Abstract

**Hintergrund:**

Untersuchungen von Krebspatienten zeigen eine moderate bis hohe Relevanz der wahrgenommenen Stigmatisierung. Bislang gibt es keine Studien, in denen Stigmatisierung in Abhängigkeit von Schmerzen bei Krebspatienten betrachtet wird. Wir analysieren den Zusammenhang von Schmerzen und Stigmatisierung bei vier häufigen Tumorentitäten.

**Methoden:**

Im Rahmen einer registerbasierten bizentrischen Studie wurden quantitative Daten von 858 Patienten (45,6 % Frauen; Altersdurchschnitt 60,7 Jahre) mit Brust‑, Darm‑, Lungen- oder Prostatakrebs ausgewertet. Die wahrgenommene Stigmatisierung wurde mit der Social Impact Scale (SIS-D) erhoben, das Instrument umfasst neben dem Gesamtscore vier Subskalen. Schmerz ist mit dem Brief Pain Inventory (BPI) erfasst worden. Die Daten wurden mittels Korrelation und multipler Regression mit soziodemografischen und medizinischen Prädiktoren analysiert.

**Ergebnisse:**

Von den 858 Krebspatienten gaben jene mit Lungen- und Brustkrebs die stärksten Schmerzen an. Die Schmerzstärke zeigt sich als Prädiktor für die Stigmatisierung bei Patienten mit Brust- und Darmkrebs. Zusätzlich kann ein jüngeres Lebensalter als Prädiktor für die Stigmatisierung festgestellt werden. Protektiv zeigt sich eine gute Lebensqualität. Die finalen Modelle weisen eine hohe Anpassungsgüte auf (korr. R^2^ > 0,35), mit Ausnahme des Regressionsmodells für Lungenkrebspatienten.

**Diskussion und Schlussfolgerung:**

Die Befunde unterstützen die Annahme eines Einflusses des Schmerzerlebens auf die wahrgenommene Stigmatisierung von Krebspatienten. Ein Zusammenhang zeigt sich zwischen der Stigmatisierung und der Depressivität. Diese Personengruppe sollte in der klinischen Praxis daher besondere psychoonkologische Aufmerksamkeit erfahren. Weitere Forschungen zu Verlauf und Mechanismen der schmerzbezogenen Stigmatisierung sind zusätzlich notwendig.

## Abstract (en)

**Background:**

Studies on cancer patients show a moderately high relevance of perceived stigmatization. However, no studies have explored the perceived stigmatization in relation to cancer-associated pain. In this work, we analysed the relationship between pain and perceived stigmatization across a large sample of four major cancer entities.

**Methods:**

Quantitative data of 858 patients (45.6% women, mean age 60.7 years) with breast, bowel, lung and prostate cancer were evaluated in a register-based, bicentric study. Perceived stigmatization was measured using the social impact cale (SIS-D), including a total score and four subscales. Pain was assessed with the brief pain inventory (BPI). The data were analysed using correlation und multiple regression with various sociodemographic and medical predictors.

**Results:**

Of all 858 cancer patients, those with lung and breast cancer were characterized by the greatest pain. The intensity of the pain was a predictor of the perceived stigma in patients with breast and colorectal cancer. In addition, younger age was also a predictor for perceived stigmatization. A good quality of life resulted as a protective factor. The final models showed a high goodness of the fit (corr. R^2^ > 0.35), except for the lung cancer patients.

**Conclusions:**

Our findings support the assumption that the experience of pain can have an impact on the perceived stigmatization of cancer patients. Depression might influence the perceived stigmatization. Therefore, this group of patients should receive special attention and psycho-oncological care in clinical practice. Further research on the course and mechanisms of action of pain-related perceived stigmatization is also required.

## Einleitung

Im Jahr 2018 erkrankten in Deutschland 265.170 Männer und 232.720 Frauen neu an Krebs, 229.065 Menschen starben an der Erkrankung. Brust‑, Prostata‑, Lungen- und Darmkrebs sind dabei die größten Diagnosegruppen und machen zusammen einen Anteil von etwa 50 % der malignen Neuerkrankungen aus [8]. Eine Krebserkrankung sowie deren Behandlung sind mit zahlreichen körperlichen und psychosozialen Krankheits- und Behandlungsfolgen verbunden [34]. Eine besonders gravierende Folge stellen krankheits- bzw. behandlungsassoziierte Schmerzen dar, nahezu jeder Patient leidet im Verlauf der Krebserkrankung unter Schmerzen. Schmerzdauer und -intensität sind von verschiedenen Faktoren abhängig. Van den Beuken et al. fanden heraus, dass zwischen 40 % (nach der kurativen Therapie) und 66 % (im fortgeschrittenen oder finalen Stadium) der Krebspatienten Schmerzen angeben [34]. Maßgeblich sind dabei direkte Tumorschmerzen, aber auch therapieinduzierte Schmerzen, die bei 7–40 % der Patienten auftreten [25]. Krebsassoziierte Schmerzen haben multiple Folgen für das Leben der Patienten. Häufig leiden sie unter einer verminderten Lebensqualität und Einschränkungen bei der Bewältigung des Alltags. Breivik et al. zeigt in einer größeren Studie, dass alltägliche Aufgaben von 69 % der Krebspatienten schmerzbedingt nicht mehr uneingeschränkt durchführbar sind und sich 52 % im Beruf beeinträchtigt fühlen, 32 % von ihnen ging es so schlecht, dass sie sterben wollten [2].

Zu den am häufigsten auftretenden Folgen von Krebs und krebsassoziierten Schmerzen zählen Depressionen, Fatigue, psychosozialer Distress sowie Angststörungen [19, 30, 36]. Verschiedene Studien haben einen Zusammenhang zwischen dem Vorkommen von Schmerzen und sozialem Rückzug bei Krebspatienten nachgewiesen [36].

Eine für Krebserkrankungen typische psychosoziale Folge ist die krankheitsbezogene Stigmatisierung. Stigmatisierung bezeichnet einen sozialen Prozess, infolgedessen Menschen aufgrund unterschiedlichster physischer oder psychischer Merkmale oder Krankheiten sozial diskriminiert und ausgegrenzt werden [13]. Goffman beschreibt Stigmatisierung als ein „körperliches oder soziales Merkmal oder Zeichen, das die soziale Identität einer Person abwertet und so dazu führt, dass es keine volle soziale Akzeptanz gibt“ [13]. Als Schutzfaktoren vor krankheitsbedingter Stigmatisierung gelten ein höheres Lebensalter, ein guter allgemeiner Gesundheitszustand, sowie ein guter psychischer Zustand. Als negative Prädiktoren gelten das weibliche Geschlecht, ein niedriger sozioökonomischer Status sowie ein reduzierter körperlicher Leistungszustand [9, 12]. Die Ausprägung der krebsbezogenen Stigmatisierung hängt von der Art der Krebserkrankung sowie von weiteren Faktoren, z. B. der Sichtbarkeit der Erkrankung (Entstellungen im Gesicht) und der subjektiv wahrgenommenen Verantwortlichkeit der Patienten für ihre Erkrankung ab [20, 27, 31]. Weiterhin hängt das Stigmatisierungserleben von interindividuell verschiedenen und subjektiv wahrgenommen Faktoren ab wie sozialer Unterstützung, eigener Resilienz und Coping-Mechanismen (z. B. Akzeptanz oder religiöse Bewältigung) [35]. Die Auswirkungen von Stigmatisierung auf Krebspatienten sind vielschichtig und zeigen sich in verminderter Lebensqualität, erhöhtem Distress und schlechterer Krankheitsbewältigung [4]. Bis dato liegen keine Befunde zum Zusammenhang von Stigmatisierung und Schmerzen bei Krebspatienten vor. Dabei lässt ein Blick auf andere Diagnosegruppen auch bei Krebspatienten einen signifikanten Zusammenhang erwarten [6, 17, 24]. In einer Untersuchung von Gichtpatienten bzw. Sichelzellanämie-Patienten zeigte sich, dass stärkere Schmerzen mit einem signifikant höheren Stigmatisierungslevel zusammenhingen (β = 0,52; *p* < 0,001 bzw. r_s_ = −0,35; *p* = 0,002) [17, 24]. Crockett et al. untersuchten den Einfluss von krankheitsbedingter Stigmatisierung auf die Schmerzstärke bei HIV-Patienten. Dabei zeigte sich, dass die Stigmatisierung im Zusammenhang mit einer erhöhten Depressivität als Mediator für eine gesteigerte Schmerzintensität wirkt [6].

Ziel der vorliegenden Studie ist es, zu zeigen, welcher Zusammenhang zwischen dem Schmerzerleben und der wahrgenommenen Stigmatisierung bei Patienten mit Brust‑, Darm‑, Prostata- oder Lungenkrebs besteht und welche soziodemografischen, medizinischen sowie belastungsbezogenen Faktoren relevant sind. Folgende Fragestellungen werden untersucht:

1. Wie stark sind die Schmerzen in der untersuchten Stichprobe in Abhängigkeit von der Diagnose ausgeprägt und wie stark hängen Schmerzen und wahrgenommene Stigmatisierung zusammen?
2. Welche Rolle spielen die Schmerzen als Prädiktor für wahrgenommene Stigmatisierung und welche weiteren soziodemografischen (Alter, Geschlecht), krankheitsspezifischen (Diagnosezeitraum, Tumorgruppe, aktueller Gesundheitszustand, Therapie) und psychosozialen Faktoren (Lebensqualität, psychosoziale Belastung, Depressivität) haben Einfluss auf die wahrgenommene Stigmatisierung von Krebspatienten?

## Material und Methoden

### Design und Datenerhebung

Im Zeitraum Mai bis September 2016 wurden im Rahmen einer Querschnittstudie und in Kooperation mit zwei klinischen Krebsregistern (Leipzig und Dresden) 1748 Patienten mit Brust‑, Prostata‑, Darm- oder Lungenkrebs postalisch kontaktiert und um Studienteilnahme gebeten. Neben den notwendigen Studienunterlagen lagen dem Schreiben ein auszufüllender Fragebogen und ein vorfrankierter Rückumschlag bei. Bei ausbleibender Antwort wurden bis zu zwei Erinnerungsschreiben versandt. Die Einschlusskriterien für die Studie waren Alter zwischen 18 und 75 Jahre, Diagnosestellung maximal 30 Monate zurückliegend, Neuerkrankung oder Rezidiv. Die Auswahl der Patienten für die Studie erfolgte geschichtet nach Diagnosegruppe, um trotz unterschiedlicher Inzidenzen etwa gleich große Substichproben zu erhalten. Ein positives Ethikvotum liegt für beide Standorte vor (Leipzig, Medizinische Fakultät der Universität Leipzig: AZ 342-15-05102015; Dresden, Universitäts KrebsCentrum: EK 442102015).

### Patienten

**Abb. 1:**
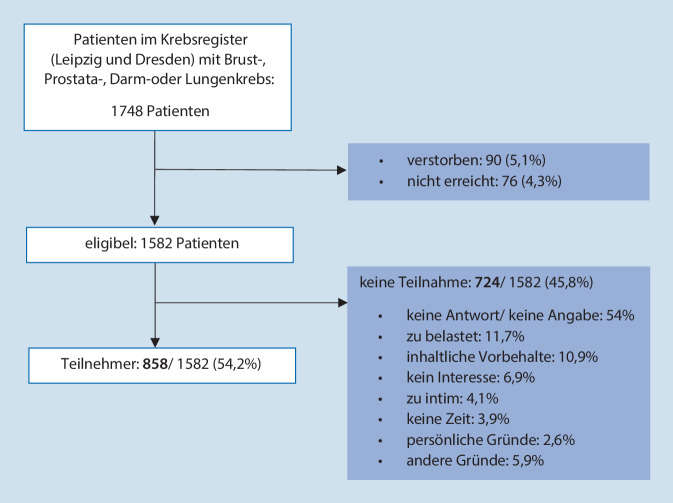
Flowchart Patientenrekrutierung

Von den 1748 kontaktierten Patienten waren 9,4 % entweder verstorben oder nicht mehr zu erreichen. Von den verbleibenden 1582 Personen (Leipzig, *n* = 696; Dresden, *n* = 886) nahmen mit einer Antwortquote von 54,2 % final 858 Patienten teil. 11,9 % lehnten eine Teilnahme wegen zu großer körperlicher oder psychischer Belastung ab und 10,9 % bzw. 6,9 % wegen inhaltlicher Vorbehalte bzw. fehlenden Interesses. Ein Drittel der 724 Nichtteilnehmer gab keinen Grund für die Nichtteilnahme an (Abb. 1).

Im Vergleich nahmen Brust- und Prostatakrebspatienten häufiger teil als jene mit Darm- und Lungenkrebs (*p* = 0,023). Patienten mit niedrigerem UICC-Stadium waren häufiger zu einer Teilnahme bereit (*p* = 0,033) (Tab. 1).

**Tab. 1:** Stichprobenbeschreibung (*n* = 858)

Merkmal		Stichprobe (*n* = 858)		Non-Responder^a^ (*n* = 724)		t/chi^2^	*p*
		*n*	%	*n*	%		
Erhebungszentrum	Leipzig	388	45,2	308	42,5	1,14	0,286
	Dresden	470	54,8	416	57,5		
Alter (Jahre)	Mittelwert (SD, Range)	60,7 (9,3; 23–73)		60,4 (9,6; 26–74)		0,26	0,607
Geschlecht	Männlich	467	54,4	402	55,5	0,19	0,685
	Weiblich	391	45,6	322	44,5		
Familienstand	Ledig	90	10,5	–	–	–	–
	Verheiratet	626	73,0				
	Geschieden	86	10,0				
	Verwitwet	54	6,3				
	Fehlende Angaben	2	0,2				
Erwerbsstatus	Erwerbstätig	360	42,0	–	–	–	–
	Rentner	426	49,7				
	Arbeitslos	20	2,3				
	Andere	24	2,8				
	Fehlende Daten	28	3,3				
Haushaltseinkommen (€/Monat)	< 2000	353	41,1	–	–	–	–
	2000–3000	266	31,0				
	> 3000	205	23,9				
	Fehlende Daten	34	4,0				
Tumorlokalisation (ICD-10)	Brust (C50)	297	34,6	220	30,4	9,52	0,023
	Colon (C26)	168	19,6	160	22,1		
	Lunge (C34)	125	14,6	139	19,2		
	Prostata (C61)	268	31,2	205	28,3		
Zeit seit Diagnose (Jahre)	Mittelwert (SD, Range)	1,9 (1,9; 0–28)		1,7 (0,75; 0–3)		3,13	0,077
–	Fehlende Daten	38	4,4	308	57,5	–	–
UICC-Stadium^b^	I	162	18,9	108	14,9^b^	8,73	0,033
	II	71	8,3	35	4,8		
	III	83	9,7	38	5,2		
	IV	47	5,5	38	5,2		
	Fehlende Daten	495	57,7	89	29,9		
Metastasen	Nein	640	74,6	–	–	–	–
	Ja	175	20,4				
	Fehlende Daten	43	5,0				
Aktuell in Behandlung	Nein	242	28,2	–	–	–	–
	Ja	565	65,9				
	Fehlende Daten	51	5,9				
Art der Behandlung (ja)^c^	Chemotherapie	367	42,8	–	–	–	–
	Radiotherapie	522	60,8				
	Operation	607	70,7				
	Hormonbehandlung	176	20,5				
	Keine Therapie	76	8,9				

^a^Aus datenschutzrechtlichen Gründen sind medizinische Informationen für Non-Responder nur für einige Variablen verfügbar

^b^Daten sind nur für Patienten aus dem Krebsregister Leipzig verfügbar

^c^Mehrfachnennungen (außer bei „keine Therapie“) möglich

## Befragungsinstrumente

Soziodemografische sowie medizinische Daten wurden im Selbstbericht erfasst bzw. mit Registerdaten ergänzt. Folgende Instrumente wurden ausgewertet:

### Schmerz

#### Brief Pain Inventory (BPI)

Zur Erfassung der Schmerzen und der dadurch bedingten Einschränkungen wurde die deutsche Version des Brief Pain Inventory genutzt [29]. Der BPI ist ein Messinstrument zur Schmerzquantifizierung bei Krebspatienten, Patienten können diesen selbstständig ausfüllen [28]. Nach zwei Screening-Fragen zur aktuellen Schmerzmitteleinnahme und Schmerzen wird die Schmerzstärke in vier unterschiedlichen Dimensionen bestimmt. Es werden die stärksten, schwächsten und durchschnittlichen Schmerzen in den letzten 24 h sowie die Schmerzen in diesem Moment auf einer Likert-Skala von 0 („keine Schmerzen“) bis 10 („stärkste vorstellbare Schmerzen“) abgefragt [29]. Zur Auswertung des Pain Severity Indexes, als Teil des BPI, können die vier Items in unterschiedliche Schweregrade eingeteilt werden. 0 bedeutet dabei „kein Schmerz“, Werte von 1–4 „milder Schmerz“, 5–6 „moderat“ und 7–10 „schwer“ [32]. Zusätzlich zur Schmerzintensität wird die Aktivitätseinschränkung der Patienten untersucht. Dafür kann der Pain Interference Index bestimmt werden. Dieser besteht aus sieben Einzelitems, welche zu einem Summenscore (Range 0–70) zusammengefasst werden können [28]. Auch hier wurde eine elfstufige Likert-Skala von 0 („keine Beeinträchtigung“) bis 10 („vollständige Beeinträchtigung“) genutzt. Bereiche möglicher Beeinträchtigungen sind „Allgemeine Aktivität“, „Normale Arbeit“ oder „Lebensfreude“.

Zur Auswertung der Items der Aktivitätsbeeinträchtigung werden die Mittelwerte der einzelnen Items genutzt. In der Validierungsarbeit zur deutschen Version liegt die interne Konsistenz Cronbachs α zwischen 0,88 (Pain Severity Index) und 0,92 (Pain Interference Index) [29]. In unserer Arbeit liegt die interne Konsistenz der beiden Indizes bei Cronbachs α = 0,93.

### Stigmatisierung

#### Social Impact Scale (SIS-D)

Zur Erfassung der wahrgenommenen Stigmatisierung wurde die deutsche Version der Social Impact Scale genutzt [7, 11, 13]. Die SIS‑D mit 24 Items ist vierfaktoriell aufgebaut. Zu den Dimensionen gehören „Soziale Isolation“ (Cronbachs α = 0,89; 9 Items, Range 0–27), „Soziale Zurückweisung“ (Cronbachs α = 0,81; 6 Items, Range 0–18), „Internalisiertes Schamgefühl“ (Cronbachs α = 0,81; 6 Items, Range 0–18) und „Finanzielle Unsicherheit“ (Cronbachs α = 0,81; 3 Items, Range 0–9). Auf einer vierstufigen Likert-Skala liegen die Antwortoptionen zwischen 0 („stimme gar nicht zu“) bis 3 („stimme voll und ganz zu“). Ein Beispielitem der Subskala Internalisiertes Schamgefühl lautet: „Ich habe das Gefühl, dass andere denken, dass ich schuld an meiner Erkrankung bin.“, ein Item der Subskala Soziale Isolation: „Ich fühle mich von den gesunden Menschen um mich herum wie abgeschnitten.“. Die insgesamt 24 Einzelitems können zu einem Gesamtscore zusammengefasst werden (Cronbachs α = 0,93) mit einer Range von 0–72 [7]. Die interne Konsistenz des Gesamtscores liegt in unserer Analyse bei α = 0,94.

### Depressivität

#### Patient Health Questionnaire (PHQ-9)

Der PHQ‑9 als Teil des Screening-Fragebogens PHQ‑D erfasst das Diagnostic and Statistical Manual of Mental Disorders (DSM-IV) Kriterien zur Diagnostik einer Major Depression. Zur Feststellung des Schweregrades der Depression wird der PHQ‑9 dimensional ausgewertet [15]. Durch die kategoriale Auswertung kann ab einem Cut-off von ≥ 10 die Diagnose der Major Depression gestellt werden (Sensitivität = 88 %, Spezifität = 88 %) [18]. Die Antwortmöglichkeiten liegen auf einer vierstufigen Skala (0 = überhaupt nicht, 3 = beinahe jeden Tag) und fragen die beschwerdebedingten Beeinträchtigungen in den letzten zwei Wochen ab. Die Bildung eines Gesamtscores von 0–27 ist möglich. Die interne Konsistenz wurde in der deutschen Validierungsstudie für den PHQ‑D mit Cronbachs α = 0,88 angegeben [15]. In unserer Untersuchung liegt sie bei Cronbachs α = 0,89.

### Lebensqualität

#### EORTC QLQ-C30

Zur Beurteilung der Lebensqualität wurde die deutsche Version des EORTC QLQ-C30 verwendet [1, 16, 17]. Es handelt sich um ein mehrdimensionales Befragungsinstrument der „European Organisation for Research and Treatment of Cancer“ (EORTC) zur Bestimmung der krankheitsassoziierten Lebensqualität bei Krebspatienten. Die insgesamt 30 Fragen umfassen neben einer Gesamtskala fünf Funktionsskalen sowie Symptomskalen und Einzelitems [1]. Wir verwendeten in der Untersuchung lediglich den Globalwert zur Lebensqualität. Die Skala rangiert zwischen 1 („sehr schlecht“) und 7 („ausgezeichnet“) und weist ein Cronbachs α von 0,86 auf [16], in der vorliegenden Untersuchung ist α = 0,93.

### Angst

#### Generalized Anxiety Disorder (GAD-7)

Der GAD‑7 ist ein Modul des Gesundheitsbogens für Patienten (PHQ-D), das der Erfassung der generalisierten Angststörung und der Einschätzung des Schweregrades der Symptomatik dient [33]. Mit sieben Items werden die wichtigsten diagnostischen Kriterien für die generalisierte Angststörung nach DSM-IV innerhalb der letzten vier Wochen erfasst. Die interne Konsistenz des Fragebogens beträgt in der Validierungsstudie Cronbachs α = 0,89 [22], in unserer Stichprobe beträgt Cronbachs α = 0,91.

### Vermeidung von Multikollinearität

Aus Gründen der Multikollinearität wurden die beiden Variablen GAD‑7 (Ängstlichkeit) sowie BPI 7 (Schmerz/allgemeine Aktivität) nicht in die Regressionsanalyse eingeschlossen. Die Variablen GAD‑7 und PHQ‑9 sowie BPI 7 und BPI 3 korrelierten in unserer Untersuchung stark mit einem Korrelationskoeffizienten r > 0,79, was auf eine große Abhängigkeit bzw. Multikollinearität hinweist und damit den Grundsatz der Unabhängigkeit der Prädiktorvariablen verletzt [10].

### Statistische Analyse

Es wird zunächst eine deskriptive Datenanalyse durchgeführt. Zur Untersuchung von Gruppenunterschieden erfolgen Mittelwertvergleiche. Zusammenhangsanalysen werden mittels Korrelation nach Pearson berechnet. Zur Darstellung multivariater Zusammenhänge zwischen dem Gesamtscore der Stigmatisierung und möglichen Prädiktoren erfolgt ein blockweiser Einschluss der Faktoren. Für jede Merkmalsgruppe (Brust‑, Darm‑, Lungen‑, Prostatakrebs) wurde ein eigenes Modell berechnet. Die Irrtumswahrscheinlichkeit ist auf 5 % festgelegt.

## Ergebnisse

Die Studienteilnehmer sind im Mittel 60,7 Jahre alt, 54,4 % sind männlich. Die Tumoren sind zu 34,6 % in der Brust, 19,6 % im Darm, 14,6 % in der Lunge und 31,2 % in der Prostata lokalisiert. Zum Zeitpunkt der Befragung befanden sich 65,9 % der Studienteilnehmer in onkologischer Behandlung (Tab. 1).

1. *Wie stark sind die Schmerzen in der untersuchten Stichprobe in Abhängigkeit von der Diagnose ausgeprägt und wie stark hängen Schmerzen und wahrgenommene Stigmatisierung zusammen?*

Tab. 2 stellt den Zusammenhang zwischen Schmerzstärke, Aktivitätseinschränkung und wahrgenommener Stigmatisierung dar. Patienten mit Lungenkrebs gaben höchste Werte für die Schmerzen (MW = 2,64) sowie für die Beeinträchtigung (MW = 2,54) an. Alle Mittelwerte befinden sich im unteren Drittel des möglichen Wertebereichs. Prostatakrebspatienten zeigen die geringsten Werte, beispielsweise liegt die Schmerzstärke bei M = 1,75 vs. M = 2,78 bei Brustkrebspatientinnen. Es zeigen sich weitestgehend mittlere und ausschließlich positive Zusammenhänge (r ≥ 0,4) mit einer Signifikanz von zumeist *p* < 0,01. Alle betrachteten Zusammenhänge sind positiv. Die höchste wahrgenommene Stigmatisierung findet sich bei Brust- (Mittelwert [MW] = 15,20) und Lungenkrebs (MW = 15,64). Unabhängig von der Tumorlokalisation zeigt sich ein starker Zusammenhang zwischen der Schmerzstärke und der Aktivitätseinschränkung (0,70 ≤ r ≤ 0,83; *p* < 0,01). Betrachtet man die wahrgenommene Stigmatisierung, findet man schwache bis mittlere Zusammenhänge zu Schmerzen mit hoher Signifikanz (0,37 ≤ r ≤ 0,44¸ *p* < 0,01).

**Tab. 2:** Interkorrelationen zwischen Schmerzen, Aktivität und wahrgenommener Stigmatisierung

Merkmale	M	SD	*n*	Stigmatisierung, gesamt	Stärkste Schmerzen
*Brust – C50*					
Stigmatisierung, gesamt	15,2	12,54	296	–	–
Stärkste Schmerzen	2,78	2,65	286	0,37**	–
Allgemeine Aktivität	2,34	2,53	287	0,42**	0,79**
*Darm – C26*					
Stigmatisierung, gesamt	13,73	12,27	167	–	–
Stärkste Schmerzen	1,53	2,15	160	0,43**	–
Allgemeine Aktivität	1,32	2	153	0,44**	0,70**
*Lunge – C34*					
Stigmatisierung, gesamt	15,64	11,39	122	–	–
Stärkste Schmerzen	2,64	2,79	116	0,30**	–
Allgemeine Aktivität	2,54	2,89	114	0,23*	0,78**
*Prostata – C61*					
Stigmatisierung, gesamt	8,4	10,25	266	–	–
Stärkste Schmerzen	1,75	2,38	258	0,38**	–
Allgemeine Aktivität	1,41	2,11	256	0,39**	0,83**

*p < 0,05

**p < 0,01

2. *Welche Rolle spielen die Schmerzen als Prädiktor für wahrgenommene Stigmatisierung und welche weiteren soziodemografischen (Alter, Geschlecht), krankheitsspezifischen (Diagnosezeitraum, Tumorgruppe, aktueller Gesundheitszustand, Therapie) und psychosoziale Faktoren (Lebensqualität, psychosoziale Belastung, Depressivität) sind von Einfluss auf die wahrgenommene Stigmatisierung bei Krebspatienten?*

Mögliche soziodemografische, krankheitsspezifische und psychosoziale Einflussfaktoren auf die wahrgenommene Stigmatisierung sind in Tab. 3 dargestellt. Sie wurden auf ihren prädiktiven Wert überprüft. In allen vier Modellen zeigt sich ein Einfluss der Depressivität auf die erlebte Stigmatisierung (*p* < 0,01). Im Modell der Brustkrebspatientinnen zeigen zudem das Alter, Chemotherapie, Schmerzstärke und Lebensqualität (*p* < 0,001) einen Einfluss auf Höhe der wahrgenommenen Stigmatisierung. Auch bei Darmkrebspatienten zeigen Schmerzstärke und Lebensqualität (*p* < 0,05) einen Effekt. Das Modell der Prostatakrebspatienten zeigt einen Einfluss des Alters (*p* < 0,05) und auch der Lebensqualität (*p* < 0,001). Im Modell der Lungenkrebspatienten findet sich neben der Lebensqualität lediglich das Geschlecht als Prädiktor für die wahrgenommene Stigmatisierung, jedoch mit geringerer Signifikanz (*p* = 0,037). Zudem zeigt es die geringste Varianzaufklärung (korr. R^2^ = 0,201). Die drei restlichen Modelle (Brust, Darm, Prostata) weisen eine hohe Anpassungsgüte auf (korr. R^2^ > 0,35).

**Tab. 3:** Prädiktoren für die wahrgenommene Stigmatisierung (Gesamtscore) von Krebspatienten (blockweiser Einschluss)

Merkmale in (): Referenzkategorie	B	SD B	Stand. β	t	*p*
*Brust – C50*					
Alter der Befragten	−0,230	0,038	−0,176	−5,988	**<** **0,001**
Hochschule (ja)	0,309	0,717	0,012	0,431	0,667
Zeit seit Diagnose	0,621	0,963	0,019	0,645	0,519
Chemotherapie (ja)	1,860	0,736	0,075	2,528	**0,012**
Stärkste Schmerzen	1,614	0,429	0,125	3,764	**<** **0,001**
Lebensqualität	−0,144	0,021	−0,252	−6,995	**<** **0,001**
Depressivität	9,378	1,025	0,311	9,146	**<** **0,001**
*Konstante*	*31,420*	*3,108*	*–*	*10,110*	***<*** ***0,001***
Erklärte Varianz (korr. R^2^)	0,379	–	–	–	–
*Darm – C26*					
Alter der Befragten	−0,017	0,076	−0,015	−0,223	0,824
Geschlecht	−0,246	1,761	−0,009	−0,14	0,889
Hochschule (ja)	0,602	1,684	0,024	0,358	0,721
Zeit seit Diagnose	−2,085	2,851	−0,048	−0,731	0,466
Chemotherapie (ja)	−0,661	1,845	−0,024	−0,358	0,721
Stärkste Schmerzen	3,296	1,092	0,224	3,019	**0,003**
Lebensqualität	−0,122	0,05	−0,19	−2,431	**0,016**
Depressivität	11,912	2,295	0,395	5,19	**<** **0,001**
*Konstante*	*20,873*	*6,884*	*–*	*3,032*	***0,003***
Erklärte Varianz (korr. R^2^)	0,387	–	–	–	–
*Lunge – C34*					
Alter der Befragten	−0,243	0,135	−0,181	−1,794	0,076
Geschlecht	−5,124	2,425	−0,21	−2,113	**0,037**
Hochschule (ja)	0,7	2,275	0,028	0,307	0,759
Zeit seit Diagnose	3,055	2,789	0,103	1,095	0,276
Chemotherapie (ja)	−0,509	2,372	−0,021	−0,215	0,830
Stärkste Schmerzen	1,734	1,249	0,152	1,388	0,168
Lebensqualität	−0,027	0,05	−0,057	−0,529	0,598
Depressivität	8,647	2,717	0,329	3,182	**0,002**
*Konstante*	*27,188*	*10,665*	*–*	*2,549*	***0,012***
Erklärte Varianz (korr. R^2^)	0,201	–	–	–	–
*Prostata – C61*					
Alter der Befragten	−0,22	0,088	−0,13	−2,503	**0,013**
Hochschule (ja)	−0,486	1,078	−0,023	−0,451	0,652
Zeit seit Diagnose	2,504	1,392	0,092	1,8	0,073
Chemotherapie (ja)	2,346	3,153	0,038	0,744	0,458
Stärkste Schmerzen	1,157	0,685	0,099	1,688	0,093
Lebensqualität	−0,159	0,033	−0,311	−4,84	**<** **0,001**
Depressivität	9,734	1,82	0,325	5,348	**<** **0,001**
*Konstante*	*28,907*	*6,042*	*–*	*4,785*	***<*** ***0,001***
Erklärte Varianz (korr. R^2^)	0,401	–	–	–	–

*B*Regressionskoeffizient B, *SD B* Standardfehler, *Stand. β* Standartisierter Regressionskoeffizient, *t* T-Wert, *p* Signifikanz

Zusammengefasst lässt sich die Schmerzstärke vor allem als Stigmatisierungsprädiktor bei Brust- und Darmkrebs feststellen. Außerdem erleben jüngere Patienten ein höheres Maß an wahrgenommener Stigmatisierung als ältere mit derselben Erkrankung. Einen protektiven Einfluss zeigt eine hohe Lebensqualität in allen untersuchten Tumorlokalisationen außer bei Lungenkrebspatienten.

## Diskussion

Die vorliegende Studie hatte zum Ziel, den Zusammenhang von Schmerzen bei Patienten mit Brust‑, Darm‑, Prostata- oder Lungenkrebs und der wahrgenommenen Stigmatisierung zu untersuchen und den Einfluss weiterer interagierender soziodemografischer, medizinischer sowie psychosozialer Variablen zu beleuchten. 858 Patienten mit Brust‑, Prostata‑, Darm- oder Lungenkrebs nahmen an der Studie teil.

Es zeigten sich unterschiedliche Schmerz- und Stigmatisierungsintensitäten bei den einzelnen Krebsarten. Lungen- und Brustkrebspatienten zeigten in unserer Arbeit die höchsten Werte für die Schmerzempfindung. Ähnliche Ergebnisse finden sich in einer Metaanalyse von Van den Beuken et al. [34]. Ursächlich dafür könnten therapieassoziierte Schmerzen wie z. B. Narbenschmerzen nach Mastektomie sein [14]. Anders als von Van den Beuken et al. beschrieben, zeigen die Darmkrebspatienten in unserer Untersuchung im Verhältnis zu den anderen Entitäten geringere Schmerzlevel. Grund dafür könnten unterschiedliche Zeitpunkte der Datenerhebung und damit auch der Therapiestadien sein. Diesen Zusammenhang zeigten Van den Beuken et al. in ihrem Review [34]. Wie auch andere Studien belegen, beschrieben die Patienten mit Prostatakrebs die geringsten Schmerzen [2]. Insgesamt liegen die Durchschnittswerte der Schmerzintensität, der Aktivitätseinschränkung sowie auch der wahrgenommenen Stigmatisierung eher im unteren Wertebereich der Skalen. Cleeland et al. zeigte in einer Untersuchung in vier Ländern Mittelwerte der Aktivitätsbeeinträchtigung von M = 5,25–6,20 [5]. In unserer Untersuchung lag der Wert etwas geringer bei M = 1,41–2,54. In die Untersuchung von Cleeland et al. wurden jedoch nur Patienten mit metastasierten Karzinomen und vorhandenen Schmerzen eingeschlossen. Außerdem erfolgte keine Angabe zur Tumorlokalisation, was die Unterschiede erklären kann [5].

Die Mittelwerte der wahrgenommenen Stigmatisierung der von uns betrachteten Krebspatienten liegen im Bereich von M = 8,4 (Prostata) bis M = 15,64 (Lunge).

Aktuell fehlen jedoch Vergleichswerte aus der Normalbevölkerung, um den Umfang der Stigmatisierung umfänglich einordnen zu können. Unsere Daten bestätigen vorherige Untersuchungen dahingehend, dass Patienten mit Lungenkrebs ein stärkeres Stigmatisierungserleben zeigen als Patienten anderer Krebsarten, was mit der unterstellten und zugeschriebenen Eigenverantwortung zusammenhänge (Krebs durch Rauchen) [3, 23].

Es zeigte sich ein positiver Zusammenhang zwischen der Schmerzintensität und der wahrgenommenen Stigmatisierung. Kleinstäuber et al. hatten dies bereits für Patienten mit Gicht berichtet [17]. Außerdem fanden Breivik et al. heraus, dass ein großer Anteil der Patienten Probleme in der Alltagsbewältigung hat [2]. Dies kann zu Rückzug und Isolation führen, wenngleich die Korrelation in unserer Untersuchung nur moderat ist. Ziel der Studie war es weiterhin, Prädiktoren für schmerzassoziierte wahrgenommene Stigmatisierung für die vier Diagnosegruppen zu identifizieren. Dabei konnten wir feststellen, dass die Schmerzstärke bei Brust- und Darmkrebs einen signifikanten Einfluss auf die wahrgenommene Stigmatisierung zeigt mit einem relativ hohen Erklärungsanteil (β). Den größten Erklärungsanteil hat in allen vier Modellen, d. h. bei allen eingeschlossenen Diagnosegruppen die Depressivität auf das Stigmatisierungserleben. Dieser Einfluss wurde auch schon für andere Erkrankungen (z. B. bei Patienten mit chronischen Schmerzen [26]) gefunden und konnte in unserer Untersuchung nun auch für Krebspatienten gezeigt werden. Aus der Literatur ist bekannt, dass stärkere Schmerzen zu erhöhtem Stress führen können [4]. Dies könnte sich maladaptiv auf die Resilienz und das Coping der Patienten auswirken.

Kroenke et al. zeigten einen Einfluss von Depressivität und Schmerzstärke auf die körperliche Aktivität und die Schwere der Behinderung der von ihnen untersuchten Krebspatienten [19], was möglicherweise zu verstärktem sozialen Rückzug führen kann und wiederum Einfluss auf die soziale Position der Patienten nimmt. Dadurch kann es zusätzlich zu einer sozialen Schwächung durch die verringerte soziale Teilhabe kommen. Wie bereits bekannt ist, ist der Stigmatisierungsprozess durch ein Stärke‑/Machtgefälle geprägt [21].

Die erklärte Varianz für die Erklärung von Stigmatisierung, die in unseren multivariaten Modellen ein zufriedenstellendes korrigiertes R^2^ zwischen 0,20 und 0,40 aufweist, ist ein Hinweis darauf, dass für die Aufklärung der untersuchten Zusammenhänge die Berücksichtigung weiterer unabhängiger Variablen notwendig ist.

### Limitationen und Stärken der Studie

Limitationen der Studie betreffen das querschnittliche Design, da hiermit keine belastbaren kausalen Zusammenhänge oder Abhängigkeiten sowie längerfristige Entwicklungen gezeigt werden können. Die Ergebnisse sind aufgrund der Einbeziehung von vier Diagnosegruppen nicht für alle Krebserkrankungen generalisierbar. Ferner wurden keine Vor- und Nebenerkrankungen der Studienteilnehmer erhoben, wodurch auch kein Einfluss dieser auf das Schmerz- und Stigmatisierungserleben geprüft werden konnte. Da sowohl Schmerzen als auch das Stigmatisierungserleben multifaktorielle Prozesse sind und sich gegenseitig bedingen, sind für die Aufklärung der Stigmatisierung weitere Faktoren (z. B. psychische und körperliche Gesundheit der Patienten) zu betrachten, welche in unserer Untersuchung aufgrund des Studiendesigns nicht erhoben wurden.

Die Stärken der Studie liegen in der Größe der Stichprobe mit Patienten der vier häufigsten Diagnosegruppen bei Krebs sowie im bizentrischen, registerbasierten Ansatz. Sie präsentiert, soweit uns bekannt, erstmalig Befunde zum Zusammenhang von Schmerzerleben mit der wahrgenommenen Stigmatisierung.

## Fazit für die Praxis

Die Ergebnisse unserer Forschung geben wichtige Hinweise auf einen moderaten Zusammenhang von Schmerzen und wahrgenommener Stigmatisierung. In der Praxis sollte deshalb auch unter diesem Aspekt auf eine gut eingestellte Symptomkontrolle bei Schmerzen geachtet werden, denn Stigmatisierung i. S. von Rückzug, Isolation oder Ausgrenzung reduziert die Lebensqualität der Betroffenen. Zusätzlich könnten Interventionen oder Unterstützungsangebote, die für Patienten mit einem höheren Stigmatisierungserleben zugeschnitten sind und auf den Abbau depressiver Symptome abzielen, nützlich sein, um das Stigma bei Krebspatienten zu reduzieren und folglich die psychische Belastung unter der Krebserkrankung und -therapie zu verringern.
